# Characterization of both myocardial extracellular volume expansion and myocyte hypertrophy by CMR in heart transplantation recipients without active rejection: implications for early cardiac remodeling

**DOI:** 10.1186/1532-429X-17-S1-O75

**Published:** 2015-02-03

**Authors:** Otavio R Coelho-Filho, Ravi Shah, Tomas G Neilan, Jose Roberto Mattos Souza, Jose Carlos Barros Júnior, Carlos Fernando R Lavagnoli, Lindemberg da Mota Silveira-Filho, Pedro Paulo Martins de Oliveira, Elaine S Severino, Otavio R Coelho-Filho, Michael Jerosch-Herold, Orlando Petrucci

**Affiliations:** 1 Medicine, State University of Campinas - UNICAMP, Campinas, Brazil; 2 Radiology, Brigham and Women's Hospital - HARVARD, Boston, MA, USA; 3 Medicine, Massachusetts General Hospital - HARVARD, Boston, MA, USA; 4 Medicine, Beth Israel Deaconess Medical Center - HARVARD, Boston, MA, USA

## Background

Left ventricular hypertrophy (LVH) after heart transplant (HTx) is multifactorial, associations include hypertension, chronic immune injury and the intrinsic effects of immunosuppression. Its consequences are significant and potentially provide an explanation for the development of diastolic dysfunction and exercise intolerance, as well as the limited life expectancy after HTx. Both expansion of myocardial extracellular volume (ECV) and myocyte hypertrophy (MH) coexist in this setting. Cardiac biopsies have limitations and may be non-representative to assess global myocardial remodeling. The goal of this pilot study was to characterize both ECV and MH by CMR in cohort of HTx recipients without active rejection.

## Methods

T1 relaxation times were measured before and after gadolinium contrast. The intracellular lifetime of water (*τic*), a cell size-dependent parameter, and extracellular volume fraction, a marker of interstitial fibrosis, were determined with a model for transcytolemmal water exchange.

## Results

Nineteen HTx recipients (mean age 50±0, 6 female, BSA 1,70±0,16m^2^, median follow-up after HTx 11±13 months) without acute rejection and 20 age matched health volunteers (mean age 51±14) underwent CMR (1.5T) includingmeasurement of LV function, T2, T1 mapping pre- and post-gadolinium and LGE, and a echocardiogram for measurement of diastolic function. HTx recipients demonstrated normal LVEF (68±11%) with a significant increased in LVMass in comparison with age-matched volunteers (LVMass 114±19g vs. 80±5g; p<0.05). Both groups (HTx and controls) did not show LGE or abnormal signal intensity in T2 images. ECV was substantially higher in HTx patients (0,43±0,14) compared with volunteers (0.29±0.03, p<0.0001). Both ECV, a marker of interstitial fibrosis, and *τic*, a new validated maker of myocyte hypertrophy, were significantly associated with LV mass (r=0.72 and r=0.68 respectively , both p<0.05). ECV and *τic* also demonstrated a strong association with E wave deceleration time (EDT) by TTE (r=0.77 and 0.74 respectively, both p<0.05). ECV maintained the positive association with EDT indexed to E wave. (r=0.66, p<0.01). by ROC curve analysis, the ECV was able to predict diastolic dysfunction using EDT by ETT with AUC 0.97 (ECV cut value 0.37, Sens 100%, Spec 86%, p<0.01).

## Conclusions

By CMR, the ECV quantification characterized expansion of extracellular volume in HTx recipients with increased LV Mass and normal LVEF. Both ECV and *τic* were associated with markers of diastolic of dysfunction after HTx. Non invasive assessment of ECV and Tau by CMR may be useful to follow HTx recipients.

## Funding

Departamental funding - State University of Campinas – UNICAMP.

